# Transformation of a low-grade follicular lymphoma into a composite lymphoma combining a high-grade B-cell lymphoma and a lymphoblastic neoplasm expressing Terminal deoxynucleotidyl Transferase: a case report

**DOI:** 10.1186/s13256-020-02433-6

**Published:** 2020-07-27

**Authors:** Antonin Bouroumeau, Eleonore Kaphan, Clémentine Legrand, Tatiana Raskovalova, Gautier Szymanski, Claire Vettier, Christine Lefebvre, Marie-Christine Jacob, Anne McLeer, Michel Peuchmaur, Rémy Gressin, Hervé Sartelet

**Affiliations:** 1 Department of Pathology, CHU de Grenoble, Grenoble, France; 2 Department of Hematology, CHU de Grenoble, Grenoble, France; 3 Laboratory of Hematology and Oncohematology, CHU de Grenoble, Grenoble, France; 4 Department of Pathology, CHU Robert Debre, Paris, France

**Keywords:** Follicular lymphoma, High-grade B-cell lymphoma, TdT, Lymphoblastic neoplasm

## Abstract

**Background:**

High-grade B-cell lymphoma with rearrangements of *MYC* and *BCL2* and/or *BCL6* is an aggressive mature B-cell neoplasm, whereas B-lymphoblastic lymphoma is immature cell proliferation, with a frequent positivity for terminal deoxynucleotidyl transferase. The transformation of a low-grade follicular lymphoma into a lymphoblastic neoplasm expressing terminal deoxynucleotidyl transferase is a very rare event.

**Case presentation:**

A 55-year-old Caucasian man was followed for a grade 1–2 follicular lymphoma carrying a t(14;18) *IGH/BCL2+* and was initially treated with R-CHOP. The follicular lymphoma presented two relapses. In the third relapse, the patient had multiple lymphadenopathy and ascites, which motivated a retroperitoneal biopsy and an ascitic tap. These samples were analyzed by histological, cytological, flow cytometric, cytogenetic, and molecular assessments. The patient died of a multiple organ dysfunction syndrome 2 weeks after his third relapse. The biopsy revealed a diffuse proliferation made up of two types of tumor cells: centroblasts (Bcl-6-positive) and immature cells (terminal deoxynucleotidyl transferase-positive). Flow cytometric analysis confirmed the immature phenotype, with an expression of terminal deoxynucleotidyl transferase, combined with a loss of membrane immunoglobulins. The cytogenetic analysis performed on the ascites revealed a clonal evolution characterized by a t(8;22)(q24;q11) *MYC+* translocation not previously detected in follicular lymphoma. Fluorescence *in situ* hybridization confirmed the double rearrangement of the *BCL2* and *MYC* genes. Polymerase chain reactions and sequencing were used to study the clonal relationship between follicular lymphoma and the secondary tumors. The *IGVH* gene rearrangement revealed a unique clonal rearrangement involving an *IGVH4–59* subset in all three specimens.

**Conclusion:**

These findings suggest a clonal relationship between the two types of lymphoma cells. Furthermore, they support the transformation of an acute follicular lymphoma into a composite lymphoma combining a high-grade B-cell lymphoma and a lymphoblastic neoplasm expressing terminal deoxynucleotidyl transferase. This case report highlights the possible transformation of follicular lymphoma into a highly aggressive and immature proliferation.

## Background

The two most common types of mature B-cell neoplasms in Western countries are diffuse large B-cell lymphoma (DLBCL) and follicular lymphoma (FL) [1]. In 5–15% of DLBCL cases, the *MYC* is rearranged [2] and frequently associated with *BCL2* or, to a lesser extent, *BCL6* translocations. These are referred to as “double-hit” or “triple-hit” lymphomas and fit the new category of high-grade B-cell lymphoma (HGBL) in the World Health Organization (WHO) updated classification system [3]. The expression of surface immunoglobulins generally indicates a mature B-cell phenotype. B-acute lymphoblastic leukemias/lymphomas (B-ALLs) are characteristically negative for surface immunoglobulins and express a phenotype of B-cell precursors, including frequent positivity for terminal deoxynucleotidyl transferase (TdT) and CD34 [4]. The association between HGBL and the expression of immaturity markers has only rarely been described. In a retrospective study, Moench *et al.* presented 13 cases of HGBL, 4 of which are characterized by the expression of TdT [5]. Furthermore, a recent study described a case of HGBL with surface light chain restriction and TdT expression [6]. The present case report describes a case involving the transformation of a low-grade FL into a composite lymphoma combining HGBL and a lymphoblastic neoplasm expressing TdT.

## Case presentation

In September 2010, a 51-year-old Caucasian man was diagnosed with multiple lymphadenopathy (clinical stage IV). His previous medical history only contained episodes of hepatitis B and C (successfully treated in 1990), and he reported no familial or psychosocial medical problems. The patient had no B symptoms but presented with a poor performance status (Eastern Cooperative Oncology Group [ECOG] 2) and a high Follicular Lymphoma International Prognostic Index score. The pathological examination performed on a lymph node biopsy established the diagnosis of a grade 1–2 FL. As a first-line treatment, the patient received six cycles of rituximab, cyclophosphamide, doxorubicin, vindesine, and prednisone (R-CHOP), followed by 2 years of rituximab maintenance. A partial response was achieved after R-CHOP and reached complete response (CR) after the first three rituximab maintenance cycles. In 2013, however, after eight rituximab cycles, new lesions appeared, with notably an enlarged cervical lymph node measuring 2 cm in diameter; at that time, the patient’s performance status was ECOG 2. A cutaneous biopsy confirmed the relapse of the grade 1–2 FL, and a second-line treatment consisting of six cycles of a bendamustine and rituximab regimen was provided. The patient again reached a CR by the end of this treatment. Eight months later, a second relapse occurred, this time with a loss of CD20 expression. Thus, a third-line treatment involving idelalisib was prescribed. However, after 3 months, this medication was determined to be responsible for interstitial pneumonitis and was therefore stopped. Two months later, the patient presented with a third progression, characterized by a severe deterioration of his performance status and the appearance of a retroperitoneal mass. In September 2015, the biopsy of this mass determined that the FL had transformed into a composite lymphoma combining HGBL and lymphoblastic neoplasm expressing TdT. The patient therefore began a fourth-line treatment, including a debulking program and the combination of cyclophosphamide, vincristine, and prednisolone. He died of multivisceral failure a few weeks later.

Immunohistochemical staining was performed using a Roche Ventana BenchMark ULTRA immunostainer (Ventana Medical Systems, Oro Valley, AZ, USA) with the following primary antibodies: BCL2 (anti-BCL2, clone 124, 1:40; Dako, Carpinteria, CA, USA), BCL6 (anti-BCL6, clone GI191E/A8, prediluted; Roche Diagnostics, Indianapolis, IN, USA), CD20 (anti-CD20cy, clone L26, 1:800; Dako), TdT (anti-TdT, polyclonal, 1:60; Cell Marque, Rocklin, CA, USA), and CD10 (anti-CD10, clone 56C6, 1:40; Cell Marque). Chromogenic detection was realized using an ultraView Universal Detection Kit (Roche).

For the cytological examination, the lymph node imprints and the ascites cytospin preparation were stained with May-Grünwald-Giemsa.

For immunophenotyping by flow cytometry (FCM), the cells extracted from the ascites fluid were first washed twice, then stained with antibodies using a direct immunofluorescence method involving erythrocyte lysis and washing. The rest of the analysis was performed using a three-laser, eight-color BD FACSCanto II TM flow cytometer and FACSDiva software (BD Biosciences, San Jose, CA, USA). The following antibodies were used: V500-CD45 (HI30; BD Biosciences), PE-Cy7-CD19 (J3-119; BD Biosciences), FITC-CD43 (1G10; BD Biosciences), PE-CD23 (EBVCS-5; BD Biosciences), PerCP-Cy5.5-CD38 (HIT2; BD Biosciences), APC-CD10 (HI10A; BD Biosciences), APC-CD22 (S-HCL-1; BD Biosciences), APC-H7-CD20 (2H7; BD Biosciences), FITC-CD44 (J.173; Beckman Coulter, Brea, CA, USA), PE-CD24 (ALB9; Beckman Coulter), FITC-TdT (HT-6; Agilent Technologies, Santa Clara, CA, USA), and FITC-Fab′_2_ rabbit antihuman kappa/PE-Fab′_2_ rabbit antihuman lambda light chains (Agilent Technologies).

Cytogenetic analysis was realized on the cell suspensions after 17 hours of unstimulated culture using R-banded metaphase chromosomes. The fluorescence *in situ* hybridization (FISH) studies were achieved using two probes (LSI *IGH/BCL2* and LSI *MYC*) according to the manufacturer’s instructions (Vysis Inc., Downers Grove, IL, United States of America (USA)).

Genomic deoxyribonucleic acid (DNA) was extracted from tumor cells according to standard procedures using a QIAamp DNA Mini Kit (Qiagen, Hilden, Germany). The clonal *IGH* rearrangements were characterized by the amplification of *IGH* variable framework regions 1, 2, and 3 (FR1, FR2, and FR3) using multiplex polymerase chain reaction (PCR) and heteroduplex analyses as previously described [7]. The PCR products were examined on 6% acrylamide gels with bands of the appropriate size.

The *IGHV-D-J* gene rearrangements were analyzed by PCR using *IGHV* family-specific FR1 primers and the antisense IGHJ primer. The positive *IGHV* products were selected for sequencing. Sequencing reactions were purified and direct sequencing was performed using the ABI PRISM 3100 Genetic Analyzer (Applied Biosystems, Foster City, CA, USA). Sequence analysis was completed using the V-QUEST international ImMunoGeneTics information system (www.imgt.org/IMGT_vquest). Output data from IMGT/V-QUEST was used to determine the *IGHV* gene use and the percentage homology to the germline.

**Fig. 1:**
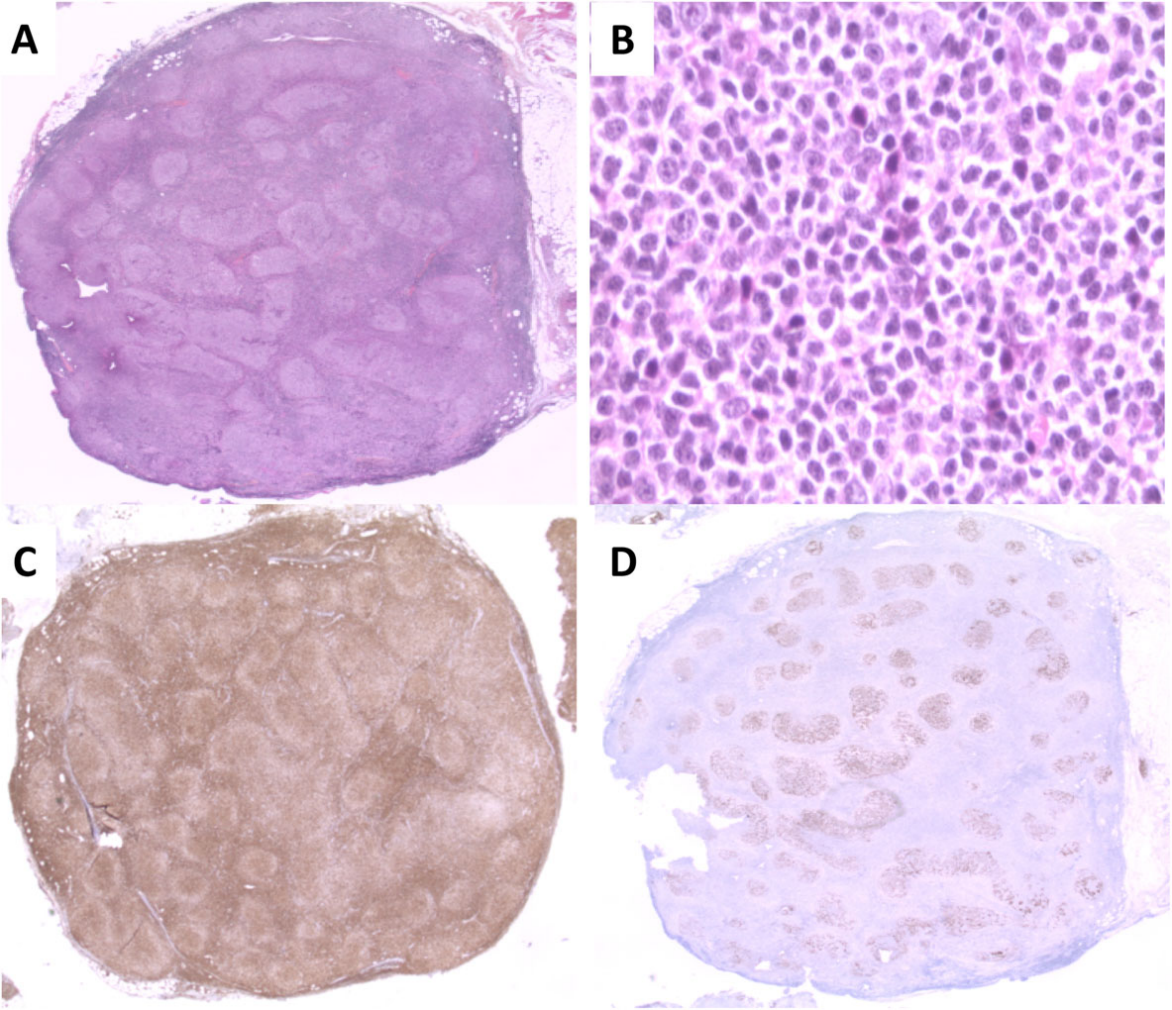
Morphological and immunohistochemical analyses of the follicular lymphoma. **a** Infiltration of the lymph node by a nodular proliferation. × 10 lens objective. **b** Nodules composed of centrocytes. × 40 lens objective. **c** Bcl-2 expression by the tumor cells. × 10 lens objective. **d** CD21 immunostaining highlighting the follicular dendritic cell meshwork. × 10 lens objective

**Fig. 2:**
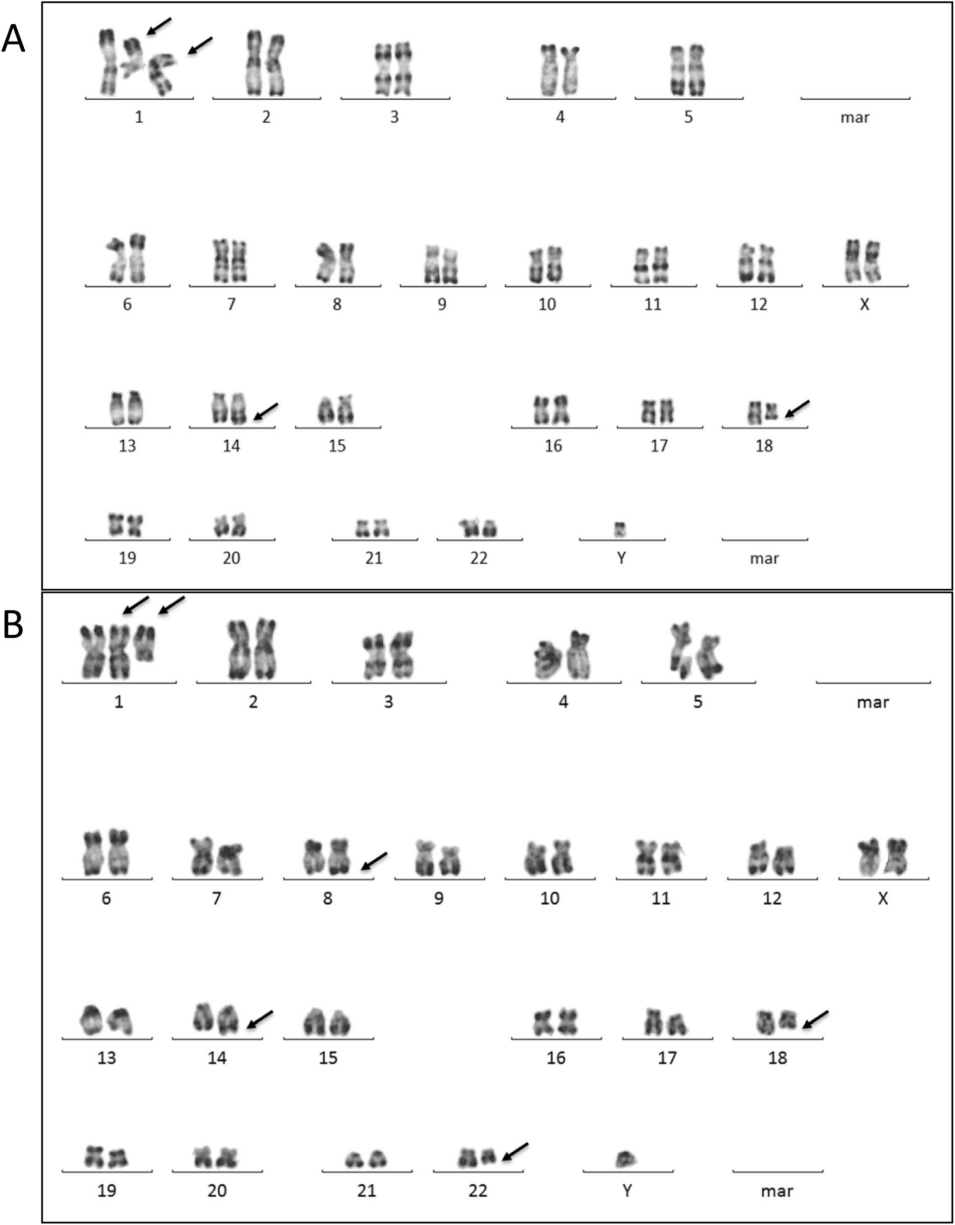
Karyotypes. **a** Karyotype performed on the first lymph node biopsy. *Black arrows* indicate the t(14;18) translocation and other cytogenetic abnormalities. Cytogenetic designation (ISCN 2016): 48,XY,+X,del(1)(q22),+i(1)(q10),t(14;18)(q32;q21), [11]/50,sl,+ 12,+ 21 [6]/46,XY [3].ish der(1)t(1;8)(q21;q24)(MYC+),8q24(MYCx2). **b** Karyotype performed on ascites fluid (third progression). *Black arrows* indicate the t(8;22) and t(14;18) translocations and other abnormalities. Cytogenetic designation (ISCN 2016): 48,XY,+X,del(1)(q21),+i(1)(q10),t(8;22)(q24;q11),t(14;18)(q32;q21) [9].ishder(1)t(1;8)(q21;q24)(5’MYC+,3’MYC+),t(8;22)(5’MYC+;3’MYC+)

**Fig. 3:**
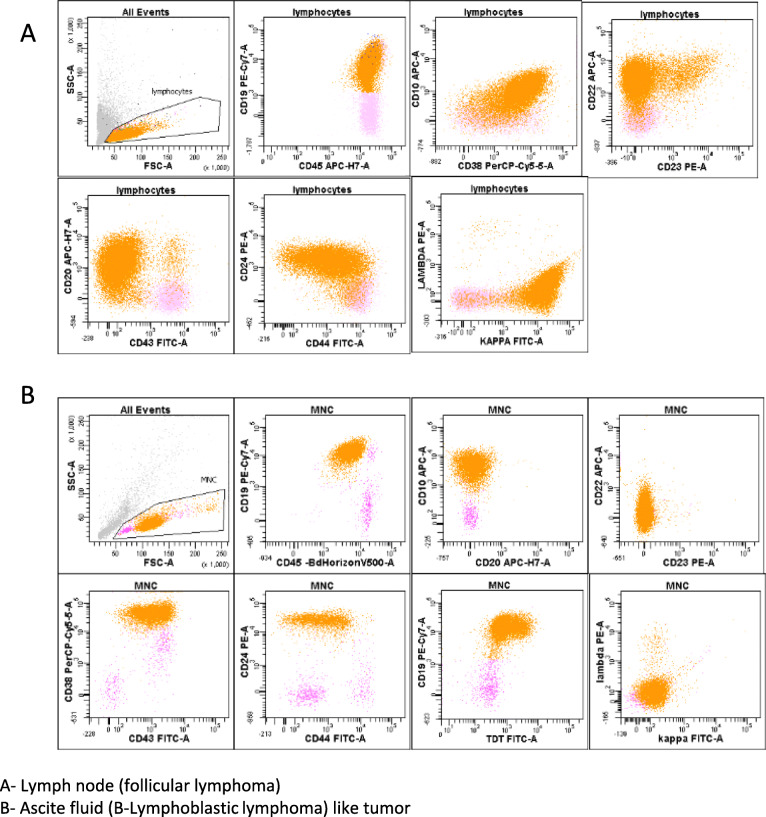
Flow cytometric analyses. **a** First lymph node (follicular lymphoma). **b** Ascites fluid (third progression) (lymphoblastic lymphoma). Flow cytometry of ascites fluid revealing a population of monotypic B-cells, expressing CD43, CD38, CD24, and terminal deoxynucleotidyl transferase, but with downregulation of pan-B markers (loss of CD20) and loss of the surface kappa light chains

**Fig. 4:**
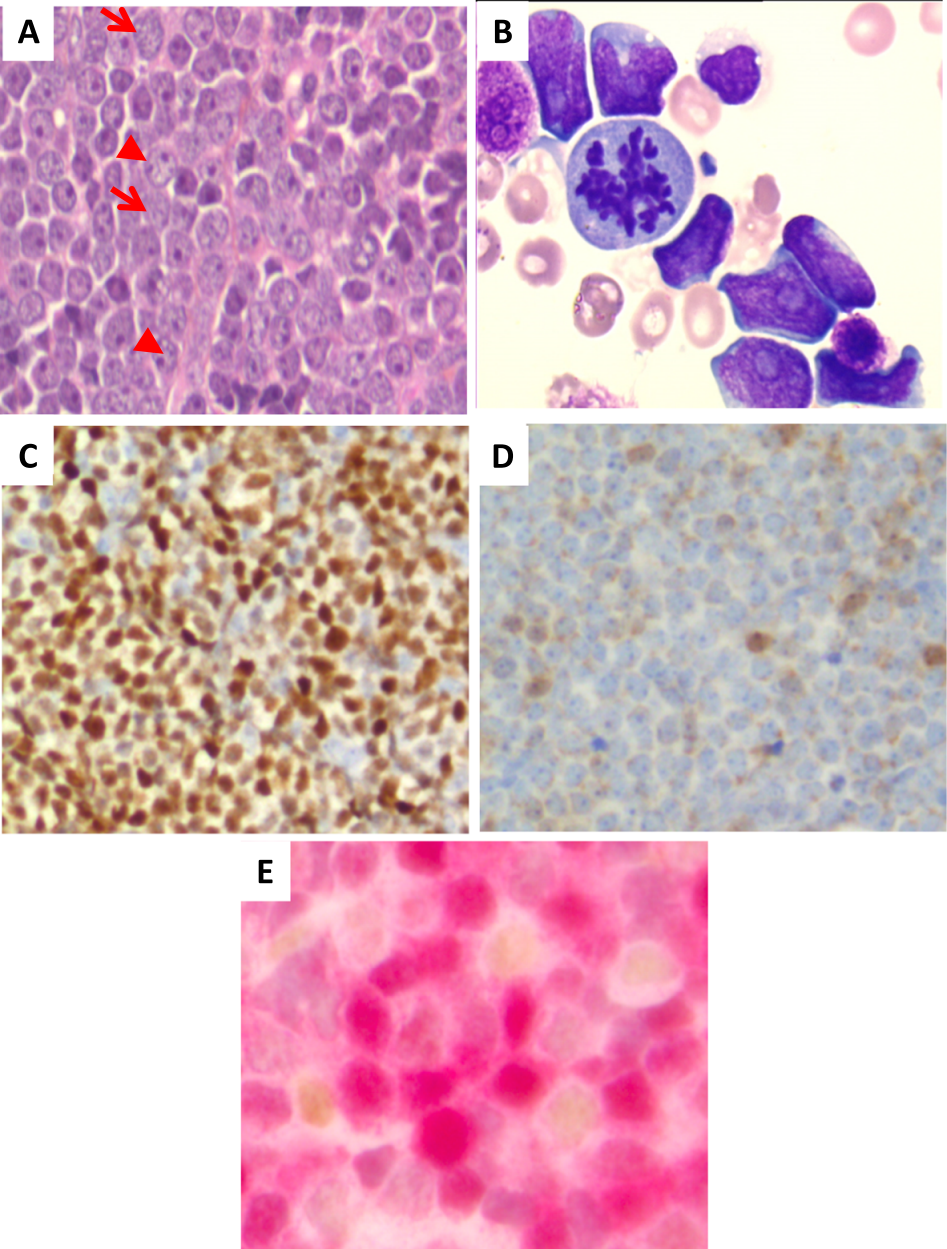
Morphological and immunohistochemical analyses of the retroperitoneal mass. **a** Lymphoblastic morphology (*arrows*) associated with centroblastic morphology (*arrowheads*), Hematoxylin and eosin stain (H&E)-stained sections, × 20 lens objective. **b** Presence of lymphoblastic cells in ascites fluid as revealed by May-Grünwald-Giemsa staining, × 100 lens objective. Presence of two populations: (**c**) one composed of centroblasts expressing Bcl-6 (× 20 lens objective) and (**d**) the other composed of lymphoblasts expressing terminal deoxynucleotidyl transferase (TdT) (× 20 lens objective). **e** Double-staining evaluation of lymphoblasts and centroblasts (TdT in brown and BCL-6 in red), × 40 lens objective

On the one hand, the first lymph node biopsy in September 2010 revealed a nodular proliferation (Fig. 1a), with predominantly centrocytic cells (Fig. 1b) expressing Bcl-2 (Fig. 1c), CD20, and CD10 as well as rare centroblastic cells. The CD21 immunostaining highlighted the follicular dendritic cell meshwork (Fig. 1d). Furthermore, the karyotype was complex and included the classical t(14;18)(q32;q21) *IGH/BCL2* translocation, as confirmed by FISH (Fig. 2a, Table 1). Moreover, FISH detected a gain of *MYC* without rearrangement (190/200 nuclei with 3 *MYC* signals) (Table 1). Finally, the FCM performed on the blood and lymph node demonstrated a high CD45, CD10^+^CD20^+^CD22^+^CD24^+^CD43^−^ population of monotypic B-cells, with surface light chain kappa restriction (Fig. 3a). On the other hand, in September 2015, the morphological and immunohistochemical analyses of the retroperitoneal mass found a diffuse lymphoid proliferation (without nodular pattern), in which a double tumor population was identified:

- Large-sized centroblast cells, which were CD20-negative, PAX-5-positive, and positive for CD10, BCL-2, BCL-6, C-MYC, and MUM1 (Fig. 4a and c)
- Blast cells with a rounded nucleus and finely mottled chromatin (Fig. 4a), which were also present in the ascites fluid (Fig. 4b) and displayed B-cell precursor markers, such as TdT expression (Fig. 4d).

**Table 1:** Cytogenetic analyses

Time of disease	Type of sample	Karyotype designation (*ISCN 2016*)	FISH Bcl2 (% of interphase cells)	FISH Myc (% of interphase cells)
Diagnosis	Lymph node	48,XY,+X,der(1)t(1;8)(q21;q14)),+i(1)(q10),t(14;18)(q32;q21), [11]/50,sl,+ 12,+ 21[6]/46,XY [3].ish der(1)t(1;8)(MYC+),8q24(MYCx2)	Rearrangement (85%)	Gain (84%)
Third progression	Ascites	48,XY,+X,der(1)t(1;8)(q21;q24),+i(1)(q10),**t(8;22)(q24;q11)**,t(14;18)(q32;q21) [9]	Rearrangement (90%)	Gain (90%) and rearrangement by t(8;22) (90%)

*FISH* Fluorescence in situ hybridization

t(8;22)(q24;q11) is associated with genomic instability and a poor prognosis

These two populations were mutually exclusive for Bcl-6 and TdT on a double-staining evaluation, confirming the presence of two cellular contingents (Fig. 4e).

The FCM performed on the ascites fluid also highlighted a population of immature B cells expressing CD43, CD10, high CD38 and CD24, and TdT. The pan-B markers CD20 and CD22 and the surface immunoglobulins were downregulated (Fig. 3b).

Moreover, cytogenetic analyses revealed a t(14;18)(q32;q21) associated with a t(8;22)(q24;q11) (Fig. 4 and Table 1). The *MYC+/BCL2+* “double hit” was validated by FISH (Table 1).

In addition, PCR and sequencing were used to study the clonal relationship between the three tumors. The PCR analysis confirmed the presence of an *IGVH* rearrangement in all specimens (Table 2). Sequence analyses revealed a unique and identical clonal *IGVH* rearrangement involving the *IGVH4–59* subset in all the samples. Moreover, the rearranged *VH* gene sequences were characterized by 85% homology with the corresponding germline sequences and therefore corresponded to somatically hypermutated *V* genes. Taken together, these data suggest that the specimens all came from a unique clonal expansion of a hypermutated *IGVH4–59* rearranged B cell.

**Table 2:** Molecular analyses

Time of disease	Type of sample	BCR clonality (IGH)	IGVH identification	Open Reading Frame	Percentage of VH homology with germline sequence	Percentage of VH homology relative to diagnosis
Diagnosis	Lymph node	FR1+,^a^ FR2-, FR3-	IGHV4–59*01, IGHD5–24*01, IGHJ4*02	Yes	87.27%	
Second progression	Lymph node	FR1+,^a^ FR2-, FR3-	IGHV4–59*01, IGHD5–24*01, IGHJ4*02	Yes	84.55%^b^	96.9%
Third progression	Ascites	FR1+,^a^ FR2-, FR3-	IGHV4–59*01, IGHD5–24*01, IGHJ4*02	Yes	86.36%	98.9%

^a^ FR1 band of the same size for the three samples

^b^ Detection of two ongoing mutations

*BCR* B Cell Receptor, *IGVH* Immunoglobulin Variable region Heavy chain, *IGH* Immunoglobin Heavy chain, *VH* immunoglobulin heavy chain variable domain

## Discussion

The accurate classification of lymphoid neoplasms is vital to determining subsequent therapy. This is why neoplasms with B-lymphoid cell lineage are broadly placed into the same category as lymphomas with a precursor B-cell phenotype and those with a mature B-cell phenotype. This classification has been extended in the latest update of the WHO guidelines, in which TdT and CD34 expression are considered exclusive for precursor B-cell neoplasms, whereas surface light chain restriction generally indicates a mature phenotype [3]. The transformation of FL into DLBCL occurs in approximately 30% of patients, but transformation into B-ALL is rare, with most cases characterized an *MYC* rearrangement in addition to an *IGH/BCL2* fusion [2, 8–15]. FL can also transform into a composite lymphoma, combining a HGBL with *BCL2* and/or *BCL6* and *MYC* rearrangements, and a B-ALL characterized by a loss of maturity markers, such as CD20 and surface immunoglobulins [2, 8–15].

As in our patient, the presence of an extra copy of *MYC* in an FL diagnosis has not previously been associated with a poor patient prognosis. A recent study of DLBCL likewise does not demonstrate a correlation between the gain of *MYC* and disease outcome [16]. Moreover, the combination of a t(14;18) with an additional copy of intact *MYC* has never been found to be associated with transformation [17].

The present report describes a case in which an initial FL subsequently transformed into a composite lymphoma characterized by an HGBL and a B-ALL-like tumor, with distinct morphological and immunophenotypic patterns. Similar findings have been reported in some recent literature on biclonal lymphomas [18, 19].

The genetic analyses in our patient’s case also demonstrate that this composite lymphoma and the initial FL have a single common ancestral clone with the same unique *IGVH4–59* rearrangement, almost the same percentage homology and the same germline *VH* sequence. Thus, the presence of a hypermutated *IGVH* strongly suggests that all the cells composing the tumor come from germinal center mature B cells. This points toward a B-lymphoblastic lymphoma-like tumor rather than a conventional B-ALL. The clonal affiliation of the follicular karyotype with the t(14; 18) of the three entities consolidates molecular data and also indicates a germinal center of origin for the B-lymphoblastic lymphoma-like tumor.

The mechanism of FL transformation in B-lymphoblastic lymphoma is not well understood, and different models have been proposed to explain its mechanism. The first model involves the “dedifferentiation” of lymphoma cells into more immature stages as a result of secondary genetic events. This is compatible with our patient’s case. The second model suggests that the two transformed neoplasms arise from a common B-cell clone, which in one case transforms into a DLBCL and in the other case “dedifferentiates” into a more immature stage. This second model is also compatible with our patient’s case. A third model proposes the presence of an additional minor undetected clone at the time of presentation, which is impossible in our patient’s case; in fact, all the clones in our patient were revealed to share a common origin.

## Conclusion

The present case report demonstrates the transformation of an FL into an HGBL with immature features, particularly TdT positivity. This particular transformation seems to be associated with a rapid progression of the disease and an unfavorable outcome.

## Data Availability

The datasets used and/or analyzed during the current study are available from the corresponding author on request.
